# How to evaluate an agent's behavior to infrequent events?—Reliable performance estimation insensitive to class distribution

**DOI:** 10.3389/fncom.2014.00043

**Published:** 2014-04-10

**Authors:** Sirko Straube, Mario M. Krell

**Affiliations:** Robotics Group, University of BremenBremen, Germany

**Keywords:** metrics, decision making, confusion matrix, oddball, imbalance, performance evaluation, classification

## Abstract

In everyday life, humans and animals often have to base decisions on infrequent relevant stimuli with respect to frequent irrelevant ones. When research in neuroscience mimics this situation, the effect of this imbalance in stimulus classes on performance evaluation has to be considered. This is most obvious for the often used overall accuracy, because the proportion of correct responses is governed by the more frequent class. This imbalance problem has been widely debated across disciplines and out of the discussed treatments this review focusses on performance estimation. For this, a more universal view is taken: an agent performing a classification task. Commonly used performance measures are characterized when used with imbalanced classes. Metrics like Accuracy, F-Measure, Matthews Correlation Coefficient, and Mutual Information are affected by imbalance, while other metrics do not have this drawback, like AUC, d-prime, Balanced Accuracy, Weighted Accuracy and G-Mean. It is pointed out that one is not restricted to this group of metrics, but the sensitivity to the class ratio has to be kept in mind for a proper choice. Selecting an appropriate metric is critical to avoid drawing misled conclusions.

## 1. Imbalance is common

In their book on signal detection theory, Macmillan and Creelman debate that comparison is the basic psychophysical process and that all judgements are of one stimulus relative to another (Macmillan and Creelman, 2004). Accordingly, many behavioral experimental paradigms are based on comparisons (mostly of two stimulus classes), like the yes–no, same-different, forced-choice, matching-to-sample, go/no-go, or the rating paradigm. When the correctness of such tasks is of interest, the overall proportion of correct responses over the two classes, i.e., the Accuracy (ACC) is the most straightforward measure. It can be easily computed and gives an intuitive measure of the performance as long as the two stimulus classes occur with equal probability. However, compared to the controlled situation in a lab where often judgements have to be made on balanced stimulus classes, natural environments provide generally different and more uncertain situations: the brain has to select the relevant stimuli irrespective of the frequency of their occurrence. Humans and animals are experts for this situation due to selection mechanisms that have been extensively investigated, e.g., in the visual (Treue, 2003) and the auditory (McDermott, 2009) domain. The behavioral relevance in a natural environment is not necessarily a matter of balance: if one is looking for an animal in the woods, the brain would have to reject many more of the irrelevant stimuli (wood) to successfully detect the relevant stimulus (animal). If the correctness of behavior concerning the two classes is estimated for such an imbalanced case, a measure like the ACC is misleading, because it is biased toward the more frequent class (Kubat et al., 1998, for discussion): missing an animal after correctly identifying many trees will not be revealed using the ACC. This is not only relevant under natural situations, but also for classical experimental paradigms, e.g., in oddball conditions which are essentially based on the fact that one class is more frequent than the other. In addition, such problems get even worse when one compares two situations with different class ratios or for dynamic situations where ratios may change over time, such as, e.g., in visual screening tasks (Wolfe et al., 2005).

To summarize, the question is how to estimate performance appropriately for imbalanced stimulus classes, i.e., which metric to use. Approaches to deal with imbalanced classes have been suggested in a number of disciplines taking different perspectives (outlined in section 2). In this broader context, a more general view of a human, animal or an artificial system will be taken in the following: an agent that discriminates incoming (stimulus) classes. Given the high number of performance measures suggested in the literature of various disciplines, the choice of an appropriate metric (or a combination) is not straightforward and often depends on more than one constraint. These constraints have to be considered carefully to avoid drawing false conclusions from the obtained metric value.

## 2. Existing approaches to deal with imbalance

Existing approaches addressing the imbalance problem can be divided into three types: modification of the underlying data, manipulation of the way the data is classified, or application of a metric that should not be affected by imbalanced classes. When the data are modified, the single instances are resampled to a balanced situation before classification or evaluation (Japkowicz, 2000; Japkowicz and Stephen, 2002; Guo et al., 2008; Sun et al., 2009; Khoshgoftaar et al., 2010). The approaches here use either oversampling of the infrequent class or undersampling of the frequent class, or a combination of both. On the classifier level, imbalance can be treated by introducing certain biases toward the infrequent class using internal modifications or by introducing cost matrices for different misclassification types. This approach is often used for artificial agents where the classification algorithm can be influenced in an explicit and formal way, e.g., by using cost-sensitive boosting (Sun et al., 2007). These two types of approaches represent the most common in the fields of machine learning, where one has full access to the training data, the test data and the classification algorithm.

However, when one does not want to re-balance the data after the experiment, the third type of approach is the most favorable for investigating the behavior of humans, animals or artificial systems. This is the typical situation in neuroscience where the behavior is investigated *as is* (within the specific scope of the experiment). Across research areas different treatments have been proposed for evaluating imbalanced classes such as genetics (Velez et al., 2007; Garcia-Pedrajas et al., 2012), bioinformatics (Levner et al., 2006; Rogers and Ben-Hur, 2009), medical data sets (Cohen et al., 2003, 2004; Li et al., 2010), data mining, and machine learning (Bradley, 1997; Fawcett and Provost, 1997; Kubat et al., 1998; Gu et al., 2008; Powers, 2011). In neuroscience, recent approaches evaluating the performance of brain-computer interfaces are trying to find a more direct and intuitive measure of performance in imbalanced cases (Zhang et al., 2007; Hohne and Tangermann, 2012; Salvaris et al., 2012; Feess et al., 2013). However, the decision for a single metric is often avoided by keeping the numbers for the two classes separated (e.g., Bollon et al., 2009; Kimura et al., 2010).

Still there is no unified concept of how to deal with this problem and which metric to choose, although this would be highly beneficial: a performance measure insensitive to imbalance enables straightforward comparisons between subjects or experiments, since individual differences in class ratio have no effect. While it is also feasible to avoid the imbalance problem by evaluating one class and ignoring the other, it bears the risk that performance qualities might be misjudged, as illustrated in section 4. An agent might yield a high performance concerning one class, but might completely fail on the other. However, in real world situations, it is equally important that the agent *accepts* the relevant signals and *rejects* the irrelevant ones. In most cases, the metric applied should directly reflect this overall behavior.

## 3. Properties of existing metrics

**Figure 1:**
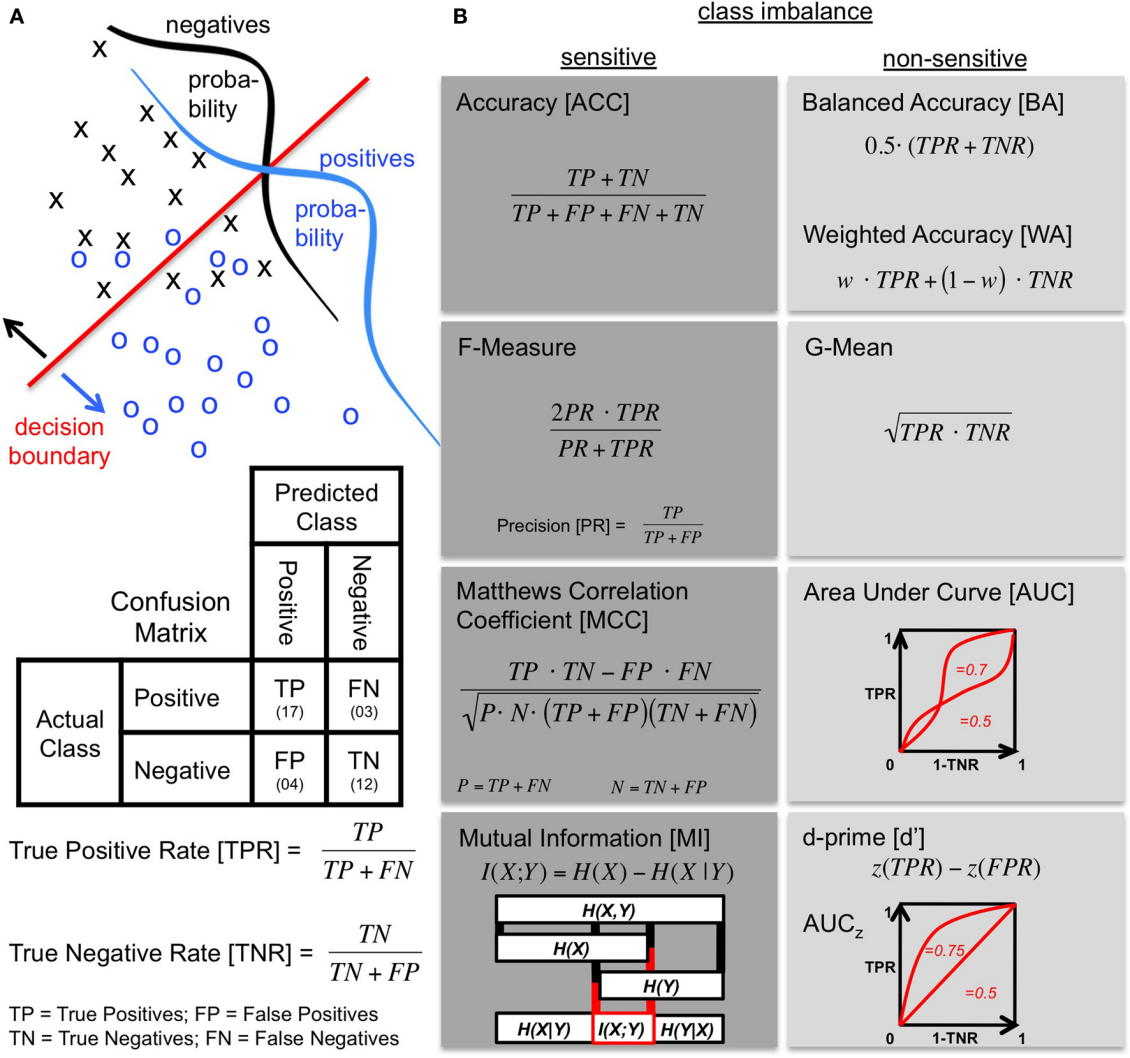
**Confusion matrix and metrics. (A)** The performance of an agent discriminating between two classes (positives and negatives) is described by a confusion matrix. Top: The probabilities of the two classes are overlapping in the discrimination space as illustrated by class distributions. The agent deals with this using a decision boundary to make a prediction. Middle: The resulting confusion matrix shows how the prediction by the agent (columns) is related to the actual class (rows). Bottom: The true positive rate (TPR) and the true negative rate (TNR) quantify the proportion of correctly predicted elements of the respective class. The TPR is also called *Sensitivity* or *Recall*. The TNR is equal to the *Specificity*. **(B)** Metrics based on the confusion matrix (see text) grouped into sensitive and non-sensitive metrics for class imbalance when both classes are considered. When the two classes are balanced, the ACC and the BA are equal with the WA being a more general version introducing a class weight *w* (for BA: *w* = *0.5*). The BA is sometimes also referred to as the *balanced classification rate* (Lannoy et al., 2011), *classwise balanced binary classification accuracy* (Hohne and Tangermann, 2012), or as a simplified version of the *AUC* (Sokolova et al., 2006; Sokolova and Lapalme, 2009). Another simplification of the AUC is to assume standard normal distributions so that each value of the AUC corresponds to a particular shape of the ROC curve. This simplification is denoted AUC_*z*_ and it is the shape of the AUC that is assumed when using the performance measure *d*′. This measure is the distance between the means of signal and noise distributions in standard deviation units given by the z-score. The two are related by $AUC_{z}=\Theta (d^{\prime }/\sqrt{2})$ where Θ is the normal distribution function. An exceptional metric is the illustrated MI, because it is based on the calculation of entropies from the confusion matrix. It can be used as a metric by computing the difference between the prior entropy H(X) determined by the class ratios and the entropy of the agent's result H(X|Y) (calculated from the confusion matrix). The boxes and connecting lines indicate the respective entropy subsets. The MI I(X;Y) is a measure of what these two quantities share.

To perform the task, the agent has some learned decision boundary to separate the two classes as is formalized in Figure 1A. Due to noise the agent labels instances to the wrong class, so that overlapping distributions with false positive (FP) and false negative (FN) decisions are obtained besides the correct ones (TP and TN). The confusion matrix comprises these four values and is the basis for most performance metrics (compare Figure 1A). Since the comparison of two matrices is difficult without a way of combining its elements, a metric is often used to compress the confusion matrix into a single number.

The choice of the metric itself heavily depends on the question addressed. Yet, this choice can be justified by certain criteria serving as guidelines: the metric should (1) evaluate the results of the agent and not the properties of the data, i.e., it should judge true performance improvements or deteriorations of the agent, (2) be as intuitive to interpret as possible, and (3) be applied such that comparisons with the existing literature remain possible. After this choice has been made, the results essentially depend on the metric properties. In extreme cases, if it has been a bad choice, another metric might lead to opposite conclusions.

Metrics that compress the confusion matrix into a single number are defined in Figure 1B. The ACC reflects the percentage of the overall correct responses and does not distinguish between the two classes. For separate handling of the two classes and thus a better approach to cope with imbalanced classes, the following two metrics have been suggested which compute the mean of the TPR and TNR. The Balanced Accuracy (BA), on the one hand, uses the arithmetic mean (Levner et al., 2006; Velez et al., 2007; Rogers and Ben-Hur, 2009; Brodersen et al., 2010; Feess et al., 2013). The G-Mean (Kubat and Matwin, 1997; Kubat et al., 1998), on the other hand, computes the geometric mean. The characteristics of the two measures differ slightly: while the BA is still very intuitive to interpret since ACC and BA are equal for balanced class ratios, the G-Mean is additionally sensitive to the difference between TPR and TNR. It has also been suggested to use different weights for TPR and TNR, so that the BA becomes a special case of the Weighted Accuracy (WA) (Fawcett and Provost, 1997; Cohen et al., 2003, 2004). The additional parameter of the WA can be used to emphasize one class during evaluation.

When the decision criterion of the agent can be influenced, the receiver operating characteristic (ROC) curve (Green and Swets, 1988; Macmillan and Creelman, 2004) is a good starting point for evaluation. It shows the performance under a varying decision criterion (Figure 1B). As a performance metric, the area under the ROC curve (AUC) is used (Swets, 1988; Bradley, 1997). Instead of comparing a single measure from a confusion matrix like the other metrics discussed here, it captures the trade-off between correct responses to both classes with the disadvantage that some decision criterion has to be varied. Calculation of this multi-point AUC is therefore not straightforward and has to be solved by numerical integration or interpolation. Two simplifications have been suggested to infer the AUC from a single data point: the interpolation of the ROC is either performed linearly which results in the same formula as the BA (Sokolova et al., 2006; Sokolova and Lapalme, 2009; Powers, 2011), or by assuming underlying normal distributions with equal standard deviations (Macmillan and Creelman, 2004). The latter approach is often used in signal detection theory and psychophysics by rating detection performance with the sensitivity measure *d*′ (Green and Swets, 1988; Stanislaw and Todorov, 1999; Macmillan and Creelman, 2004). Each value of *d*′ corresponds to one specific ROC curve with area *AUC*_*z*_ (see Figure 1B).

In contrast to ROC analysis, computation of the F-Measure (Rijsbergen, 1979; Powers, 2011) only requires three numbers from the confusion matrix (TP, FN and FP), because with the F-Measure one is solely interested in the performance on the positive class. It is often used in information retrieval when the negative class is not of interest, e.g., because the TNs cannot be determined easily. In this respect, it has been suggested as a metric for imbalanced classes. As indicated in Figure 1B, the F-Measure combines the TPR with the proportion of all positive classifications that are correct, called precision (PR) or positive predictive value, using the harmonic mean of the two. Similar to the geometric mean, the harmonic mean is sensitive to differences of its entities.

An attempt to infer the goodness of performance from the correlation between the true class labels and the agent's decisions is provided by Matthews Correlation Coefficient (MCC). The MCC (also known as phi correlation coefficient) comes from the field of bioinformatics (Matthews, 1975; Gorodkin, 2004; Powers, 2011) and evaluates the Pearson product-moment correlation between the true labels and the classification outcome. For computation of the MCC, the two classes are not handled independently, as one can see from the equation in Figure 1B.

Finally, the quantification of mutual information (MI) is, like the MCC, an attempt to compare the true world with the agent's decision. The difference is in the concept: MI, denoted by I(X;Y), is based on the comparison of information content measured in terms of entropy. The entropy of the true world is the prior entropy H(X) which is solely computed from the ratio between the two classes. The agent predicts H(X|Y) (calculated from the confusion matrix) using his own entropy H(Y). MI is a measure of what the classification result and the true class distribution have in common (compare Figure 1B). It is often used in neuroscience to characterize the quality of neural responses (Pola et al., 2003; Quiroga and Panzeri, 2009; Smith and Dhingra, 2009) or has been suggested for the prediction of time series (Bialek et al., 2001). As a performance measure, MI has been suggested for discrimination tasks as a tool to complement classical ideal observer analysis (Thomson and Kristan, 2005) and to evaluate classification performance (Metzen et al., 2011). Since the raw value obtained for MI is depending on the prior entropy H(X) (determined from the class ratio), it is straightforward that MI values for different class ratios should be compared using a normalized MI (nMI) (Forbes, 1995).

## 4. Different metric—different result

**Figure 2:**
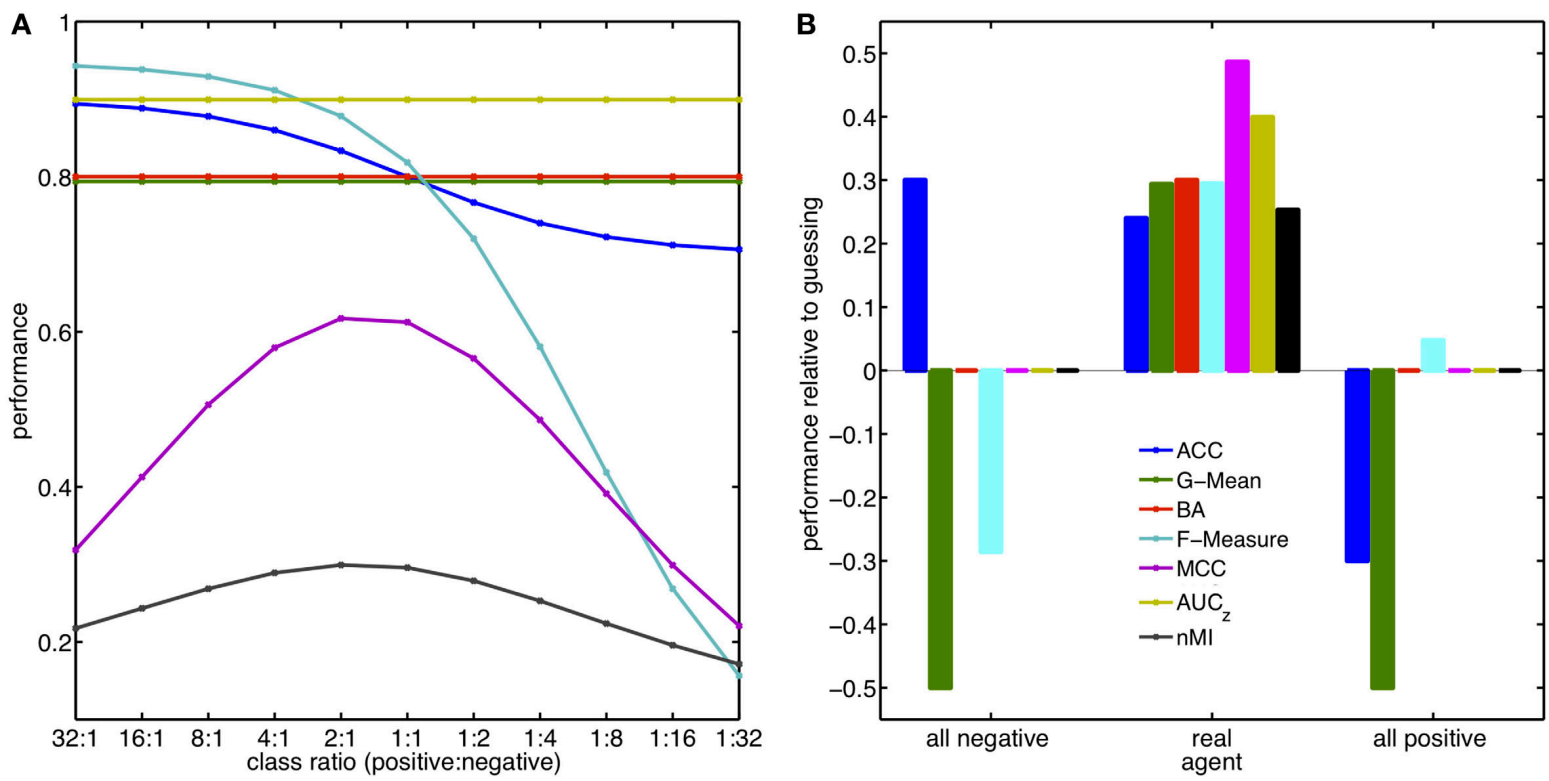
**Performance, Class Ratios, and Guessing.** Examples of metric sensitivities to class ratios **(A)** and agents that guess **(B)**. Effect of the metrics AUC and *d*′ are represented by AUC_*z*_ using the simplification of assumed underlying normal distributions. The value for *d*′ in this scenario is 0.81. Similarly, the BA also represents the effect on the WA. **(A)** The agent responds with the same proportion of correct and incorrect responses, no matter how frequent positive and negative targets are. For the balanced case (ratio 1:1) the obtained confusion matrix is [TP 90; FN 10; TN 70; FP 30]. **(B)** Hypothetical agent that guesses either all instances as positive (right) or as negative (left) in comparison to the true agent used in **(A)**. Class ratio is 1:4, colors are the same as in **(A)**. The performance values are reported as difference to the performance obtained from a classifier guessing each class with probability 0.5, i.e., respective performances for guessing are: [ACC 0.5; G-Mean 0.5; BA 0.5; F-Measure 0.29; MCC 0; AUC_*z*_ 0.5; nMI 0].

The outcome of a study should not be affected by an improper choice of the metric. Here, the sensitivity of the described metrics to class imbalance is illustrated with two examples that can be easily reproduced. In the first example, it is mimicked that a task has been performed and the investigator ends up with a confusion matrix and has to judge a performance. It is assumed that the agent performs with the same proportion of correct and incorrect responses irrespective of the ratio between the classes (TPR = 0.9; TNR = 0.7). Therefore, the agent would obtain twice as many TPs and FNs, when, the occurrence of the positive class is doubled. The metrics introduced in section 3 were used to estimate the performance for each of the different class ratios applied. Sensitivities of these metrics to changes in the underlying class ratio are depicted in Figure 2A. ACC, F-Measure, MCC and MI behave sensitive to the introduced imbalance, because they are not built from a separate evaluation of the two classes. By contrast, G-Mean, BA (WA) and AUC (*d*′) stay constant revealing what actually happened: the agent did not change its behavior. This example illustrates how important it is to carefully select the metric with respect to the data.

The second example illustrated in Figure 2B takes a different perspective. What happens to the value of the respective metric when the class ratio is fixed, but the agent changes its strategy to the extreme case of responding solely with one class no matter which data it received? To illustrate this, the same confusion matrix as in the first example was used and the class ratio fixed to 1:4. The performance changes relative to pure guessing (TPR = TNR = 0.5) are computed for an agent labeling all instances as negative or positive, respectively. Most metrics show what should be revealed: the modified agent is not better than guessing. However, the values obtained for ACC, F-Measure and G-Mean show a deviation from guessing. Most misleading is the obtained ACC of 0.8 for the case where all instances were classified as negative. This indicates a meaningful decision of the agent, and, yet, the ACC is purely based on the fact that the negative instances are four times more frequent. Even worse, the estimated performance of this failing agent is better than the one of the real agent (0.74).

## 5. Conclusions: metrics insensitive to imbalanced classes

Many treatments to the imbalance problem have been suggested, but only some of them are applicable when one wants to evaluate the behavior of an agent that cannot be changed and comes *as is*, like it is often the case in neuroscientific studies. Then, the influence of different class ratios can be minimized by two approaches: either one can re-balance the data afterwards with the drawback of neglecting the true distributions in the task, or a metric can be chosen which is largely insensitive to the imbalance problem. The variety of used metrics makes this choice not straightforward. As has been illustrated, some metrics like the ACC are highly sensitive to class imbalance, while others like the BA are not. More generally, it appears that a reliable choice for imbalanced classes is a metric that separately treats positive and negative class as TPR and TNR, like WA, BA, G-Mean, *d*′, and AUC. Out of these, the BA is probably the most intuitive, because it can be interpreted similar to the ACC as a *balanced* percent correct measure. For the more general WA the respective weights have to be fairly determined, so if the two classes are equally important the BA is a proper choice.

Despite the fact that the situation is more complicated when more than two classes are considered, some of the principles illustrated here remain useful. Although the transfer of the suggested metrics to a multi-class scenario is not straightforward, it still holds that metrics that equally treat the existing classes as performance rates are robust to changes in the individual class ratios. In addition, it would be favorable if the value of the metric is independent of the number of classes, such that, e.g., the same metric value in two experiments with different numbers of classes refers to the same performance. For the BA in an experiment with m classes, this could be achieved by summing up all m rates and dividing them again by m. As an alternative approach, many multi-class problems can be boiled down to a two-class problem for evaluation, e.g., by dividing the individual class examples into relevant and irrelevant before evaluation.

Finally, it should be stressed that the purpose of this review is to outline the implications when using imbalanced classes, and not to render metrics as generally inappropriate. Finding an appropriate metric for a particular question is complicated and often multiply constrained. Sometimes it may be necessary to use multiple metrics to complete the picture. When choosing a metric, one has to be aware of its particular drawbacks to know the weaknesses of one's own analysis. This is of critical importance, because the applied metric is the basis for all performance judgements in the respective task. Therefore, it should be informative, comparable and concurrently give an intuitive access for better interpretability. For imbalanced classes it is difficult to compare values of a metric where the guessing probability is depending on the class ratio, like is the case for the F-Measure. To generally improve the comparability between studies, the confusion matrix and an estimate of the class distribution could be supplementarily reported to the metric used. Many performance metrics can be computed from these numbers, so reporting these numbers could serve as a common ground to compare one's own results to existing ones even if a different metric was chosen. This information could be provided in a compressed way, e.g., the BA and the TPR alone can be used to compute a confusion matrix (containing rates).

### Conflict of interest statement

The authors declare that the research was conducted in the absence of any commercial or financial relationships that could be construed as a potential conflict of interest.
